# Methotrexate administration directly into the fourth ventricle in children with malignant fourth ventricular brain tumors: a pilot clinical trial

**DOI:** 10.1007/s11060-015-1878-y

**Published:** 2015-08-09

**Authors:** David I. Sandberg, Michael Rytting, Wafik Zaky, Marcia Kerr, Leena Ketonen, Uma Kundu, Bartlett D. Moore, Grace Yang, Ping Hou, Clark Sitton, Laurence J. Cooper, Vidya Gopalakrishnan, Dean A. Lee, Peter F. Thall, Soumen Khatua

**Affiliations:** Division of Pediatric Neurosurgery, Departments of Pediatric Surgery and Neurosurgery, University of Texas Health Science Center at Houston and Mischer Neuroscience Center, 6431 Fannin Street, MSB 5.144, Houston, TX 77030 USA; Divisions of Neurosurgery and Pediatrics, University of Texas MD Anderson Cancer Center, Houston, TX USA; Division of Pediatrics, Unit 87, University of Texas MD Anderson Cancer Center, 1515 Holcombe Blvd., Houston, TX 77030 USA; Division of Neurosurgery, Department of Pediatric Surgery, University of Texas Health Science Center at Houston, 6431 Fannin St., MSB 5.146, Houston, TX 77030 USA; Unit 1482, Department of Diagnostic Imaging, Section of Neuroradiology, FCT 16.5020, University of Texas MD Anderson Cancer Center, 1400 Pressler Street, Houston, TX 77030 USA; Unit 85, Department of Pathology, University of Texas MD Anderson Cancer Center, 1515 Holcombe Blvd., Houston, TX 77030 USA; Unit 1472, Department of Imaging Physics, University of Texas MD Anderson Cancer Center, 1515 Holcombe Blvd., Houston, TX 77030 USA; Department of Diagnostic & Interventional Imaging, University of Texas Health Science Center at Houston, 6431 Fannin Street, MSB 2.130B, Houston, TX 77030 USA; Ziopharm Oncology Inc., 1 First Avenue; Parris Building, #34, Navy Yard Plaza, Boston, MA 02129 USA; Department of Biostatistics, Office FCT 4.614, MD Anderson Cancer Center, Houston, TX 77230-1402 USA

**Keywords:** Fourth ventricle, Intraventricular chemotherapy, Medulloblastoma, Ependymoma, Methotrexate

## Abstract

We hypothesize that chemotherapy can be safely administered directly into the fourth ventricle to treat recurrent malignant brain tumors in children. For the first time in humans, methotrexate was infused into the fourth ventricle in children with recurrent, malignant brain tumors. A catheter was surgically placed into the fourth ventricle and attached to a ventricular access device. Cerebrospinal fluid (CSF) flow was confirmed by CINE MRI postoperatively. Each cycle consisted of 4 consecutive daily methotrexate infusions (2 milligrams). Disease response was monitored with serial MRI scans and CSF cytologic analysis. Trough CSF methotrexate levels were sampled. Five patients (3 with medulloblastoma and 2 with ependymoma) received 18, 18, 12, 9, and 3 cycles, respectively. There were no serious adverse events or new neurological deficits attributed to methotrexate. Two additional enrolled patients were withdrawn prior to planned infusions due to rapid disease progression. Median serum methotrexate level 4 h after infusion was 0.04 µmol/L. Range was 0.02–0.13 µmol/L. Median trough CSF methotrexate level 24 h after infusion was 3.18 µmol/L (range 0.53–212.36 µmol/L). All three patients with medulloblastoma had partial response or stable disease until one patient had progressive disease after cycle 18. Both patients with ependymoma had progressive disease after 9 and 3 cycles, respectively. Low-dose methotrexate can be infused into the fourth ventricle without causing neurological toxicity. Some patients with recurrent medulloblastoma experience a beneficial anti-tumor effect both within the fourth ventricle and at distant sites.

## Introduction

Despite improved outcomes for patients with medulloblastoma and other malignant posterior fossa tumors over the past few decades, survival rates remain low for patients with recurrence following initial surgical resection and adjuvant therapy [1]. Moreover, current treatment regimens for relapsed malignancies can cause considerable morbidity. Novel approaches are warranted in order to improve survival rates and minimize toxicity. This pilot clinical trial is based upon the hypothesis that infusion of chemotherapeutic agents directly into the fourth ventricle of the brain may optimize local and regional drug concentrations and improve tumor control rates.

This study marks the first trial in humans of chemotherapy administration into the fourth ventricle. Our primary objective was to demonstrate that chemotherapy may be safely infused into the fourth ventricle without causing new neurological deficits or evidence of damage to the adjacent brainstem or cerebellum on magnetic resonance imaging (MRI) scans. Our secondary objective was to assess if methotrexate infusions into the fourth ventricle, without any simultaneous systemic chemotherapy, had any measurable disease response.

## Materials and methods

This study (clinicaltrials.gov ID NCT01737671) was performed in accordance with the ethical standards as outlined in the 1964 Declaration of Helsinki and its later amendments. The study was initiated after IND exemption was obtained from the FDA (IND Exemption Number 116804) and institutional review board approval was granted by the University of Texas MD Anderson Cancer Center (Protocol 2012-0823) and Children’s Memorial Hermann Hospital/University of Texas Health Science Center at Houston (Protocol HSC-MS-12-0492). All patients and/or their parents or legal guardians signed informed consent.

Eligible patients included children ≤21 years of age with medulloblastoma, ependymoma, or atypical teratoid/rhabdoid tumor (ATRT) that originated in the posterior fossa and subsequently recurred anywhere in the brain and/or spine. Patients were excluded if they were pregnant, receiving any other chemotherapy, or enrolled in another experimental protocol. An estimated life expectancy of at least 12 weeks and a Karnofsky or Lansky performance score of 50 or greater was required [2].

**Fig. 1:**
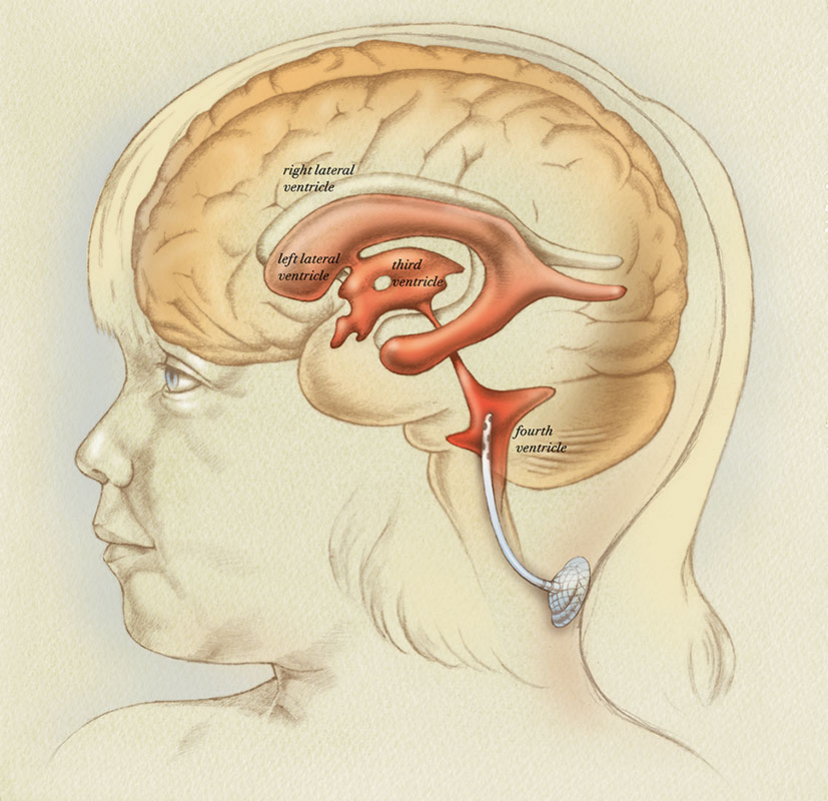
Artist’s illustration demonstrating surgical placement of catheter into the fourth ventricle of the brain attached to subcutaneous reservoir

Enrolled patients underwent a posterior fossa craniotomy and maximal safe surgical resection if resectable tumor was within the operative field. A ventricular catheter was then placed under direct vision into the fourth ventricle. The dura mater was closed in a water-tight fashion around the catheter, and the catheter was attached to a ventricular access device (VAD) which was implanted subcutaneously at the inferior aspect of the incision (Fig. 1).

**Fig. 2:**
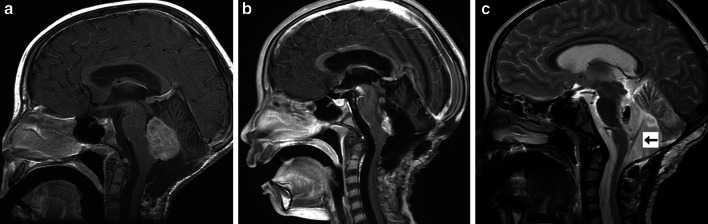
Preoperative and postoperative MRI images from patient 2. **a** Preoperative sagittal T1-weighted MRI with gadolinium demonstrating recurrent, enhancing tumor filling fourth ventricle. **b** Postoperative sagittal T1-weighted MRI with gadolinium demonstrating subtotal resection of tumor. A small amount of tumor adherent to the floor of the fourth ventricle was purposefully left behind. **c** Postoperative T2-weighted sagittal MRI demonstrating catheter position within the fourth ventricle. The catheter, as shown by the *arrow*, is positioned in a trajectory such that injury to the brainstem and cerebellum are avoided and all catheter holes are within the fourth ventricle

Postoperatively, patients underwent an MRI of the brain with gadolinium to assess both baseline tumor burden and catheter placement within the fourth ventricle (Fig. 2). Additionally, CINE MRI sequences of the brain and total spine were performed to confirm CSF flow from the fourth ventricular outlets to the cervical, thoracic, and lumbar spine. CSF flow was characterized as present or absent by the study neuroradiologist. If CSF flow from the fourth ventricle to the lumbar spine was not definitively present, then a nuclear medicine CSF flow study with Indium-111 diethylenetriaminepenta-acetic acid (In-111 DPTA) was required prior to methotrexate infusions. Patients additionally underwent lumbar puncture to assess CSF cytology at least 10 days postoperatively and before initiation of chemotherapy.

Intraventricular methotrexate cycles were initiated at least 2 weeks after VAD placement. All patients were admitted to the hospital for the first 3 cycles, and subsequent cycles were performed on an outpatient basis. Neurological examinations were performed immediately before and after each infusion. Each cycle consisted of four consecutive daily infusions of methotrexate (2 mg prepared in 2 mL of preservative-free normal saline followed by a 1 mL flush of preservative-free normal saline). Prior to each infusion, 3 mL of CSF was aspirated from the VAD for trough methotrexate level, cytology, gram stain and culture, cell count, glucose and protein. Serum methotrexate levels were measured 4 h after each methotrexate infusion for at least the first 3 cycles. Serum and CSF methotrexate levels were measured using previously reported fluorescence polarization immunoassay techniques [3]. Leucovorin rescue (5 mg/m^2^ per dose) was planned if any serum methotrexate level was >0.3 µmol/L. Patients did not receive any simultaneous systemic chemotherapy.

An MRI scan of the brain was obtained within 48 h after completion of the first cycle primarily to identify new signal changes in the brain after methotrexate infusions. MRI scans of the brain and/or spine to assess disease response were obtained after the third cycle and after every third cycle thereafter until therapy was stopped. Disease response was assessed by comparing MRI scans after surgery (pre-treatment baseline) with MRI scans obtained after every subsequent three cycles. Lumbar puncture to assess CSF cytology was repeated after the third cycle and subsequently after every additional three cycles if the prior lumbar puncture showed positive cytology. Table 1 outlines criteria used for assessing disease response. Criteria for stopping therapy included either disease progression or the judgment of the patient’s treating physicians that maximal benefit had been achieved. All patients underwent neuropsychological evaluation with age-appropriate test batteries postoperatively before fourth ventricular chemotherapy administration and again after completion of the third cycle.

**Table 1:** Assessment of disease response

**Selection of target and non-target lesions**
*Target lesions* All measurable lesions were defined as “target” lesions. If multiple measurable lesions were present, up to five were selected as target lesions based upon size and suitability for accurate repeated measurements. Lesions were measured in whichever MRI sequence (i.e. T1, T2, FLAIR, etc.) which provided the most accurate measurement of tumor size
*Non*-*target lesions* Included lesions which could not be measured accurately, such as leptomeningeal disease. CSF cytology (e.g. CSF positive or negative for tumor cells) was followed as a non-target lesion
**Response criteria for target lesions**
Target lesions were measured in 2 dimensions: the longest diameter and perpendicular to the longest diameter. Response criteria were assessed based on the product of the longest diameter and its longest perpendicular diameter
*Complete response (CR)* No evidence of disease
*Partial response (PR)* ≥50 % decrease in the sum of the products of the two perpendicular diameters of all target lesions (up to 5)
*Stable disease (SD)* Neither sufficient decrease in sum of the products of the two perpendicular diameters of all target lesions to qualify for PR nor sufficient increase in a single target lesion to qualify for PD
*Progressive disease (PD)* ≥25 % increase in the product of perpendicular diameters of any target lesion or the appearance of any new lesions
**Response criteria for non-target lesions**
*Complete response (CR)* Disappearance of all non-target lesions
*Stable disease (SD)/incomplete response (IR)* The persistence of one or more non-target lesions
*Progressive disease (PD)* The appearance of one or more new lesions and/or unequivocal progression of existing non-target lesions

**Overall response assessment**			
Target lesions	Non-target lesions	New lesions	Overall response
CR	CR	No	**CR**
CR	IR/SD	No	**PR**
PR	CR, IR/SD	No	**PR**
SD	CR, IR/SD	No	SD
PD	Any	Yes or no	**PD**
Any	PD	Yes or no	**PD**
Any	Any	Yes	**PD**

Overall Response Assesment values are in bold

## Results

Seven patients with a median age of 12 years (range 4–19 years) were enrolled (Table 2). Five patients had recurrent medulloblastoma, and two had recurrent anaplastic ependymoma. All seven patients had progressive disease at the time of enrollment despite prior surgery, radiation therapy, and various chemotherapy regimens. Two patients (patients 1 and 3) had received prior intravenous methotrexate, both at least 3 years prior to enrollment.

**Table 2:** Patient data and treatment responses

Patient^a^	Age/sex	Diagnosis	Recurrent disease sites at time of enrollment	Extent of resection^b^	# of cycles	Treatment response^c^
1	19/M	Medulloblastoma	4th ventricle, cerebellar folia, posterior fossa cisterns, supratentorial, spine	Biopsy	18	SD
2	9/M	Anaplastic Ependymoma	4th ventricle, leptomeninges of brainstem and cervical spine	Subtotal resection	9	SD
4	12/M	Medulloblastoma	Spinal subarachnoid space	None	12	PR
5	16/F	Medulloblastoma	Right and Left lateral ventricles	None	18	PR
7	8/M	Anaplastic Ependymoma	4th ventricle	Subtotal resection	3	PD

*SD* Stable disease, *PR* partial response, *PD* progressive disease, *N/A* not applicable

^a^Patients 3 and 6 are not included in this Table because they were enrolled and underwent fourth ventricle catheter/reservoir placement but never received treatment

^b^In addition to fourth ventricle catheter/reservoir placement

^c^Best treatment response

All patients underwent successful surgical implantation of the catheter and VAD. Simultaneously, three patients underwent subtotal resection of tumor located in the fourth ventricle or cerebellum, and one patient underwent simultaneous tumor biopsy. The remaining three patients did not have disease in the posterior fossa amenable to simultaneous resection. Six patients had no new neurological deficits after surgery, and one patient (patient 7) had partial left-sided facial weakness postoperatively. This patient had undergone simultaneous subtotal resection of tumor (ependymoma) that was filling the fourth ventricle. The post-operative seventh nerve palsy was attributed to resection of tumor close to the floor of the fourth ventricle and did not resolve for the duration of follow-up. Post-operative MRI scans in all patients demonstrated catheter placement in the fourth ventricle without injury to the brainstem or cerebellum (Fig. 2). In all patients, CINE MRI sequences confirmed CSF flow from the fourth ventricular outlets to the cervical, thoracic, and lumbar spinal subarachnoid spaces. Nuclear medicine CSF flow studies were therefore not required in any patients.

Two patients (patients 3 and 6) underwent surgery for VAD implantation but never received methotrexate infusions. Both of these patients were discharged from the hospital postoperatively with no new neurological deficits. In the interval between surgery and the planned first cycle, both patients had rapid disease progression. Patient 3 presented with irritability and decreased strength in his arms and legs 2 weeks postoperatively. A new MRI scan demonstrated massive hydrocephalus caused by widespread worsening of leptomeningeal tumor throughout the brain as well as tumor progression in the spine causing spinal cord compression. A palliative ventriculoperitoneal shunt was placed, and he was taken off study without ever receiving methotrexate infusions. He died 10 days later. Similarly, patient 6 presented 7 days postoperatively with swallowing difficulty and facial asymmetry followed by vomiting and lethargy. An MRI demonstrated massive tumor progression throughout the brain. She was taken off study and died 2 days later.

The five patients who underwent methotrexate infusions received 18, 18, 12, 9, and 3 cycles respectively, totaling 240 infusions. There were no new neurological deficits after these infusions either immediately or subsequently for the duration of follow-up (range 4–24 months from first infusion, median = 13 months). One patient (patient 1) had significant improvement in prior neurological deficits including improved gait, decreased dysmetria, and decreased nystagmus over the course of treatment. One patient (patient 4) had intermittent grade 3 abdominal pain which occurred during 4 of 12 cycles and lasted up to 2 days after the completion of these cycles. He underwent extensive work-up including abdominal plain X-rays, CT scan, endoscopy, and colonoscopy, all of which were negative. Because this patient had a ventriculoperitoneal shunt, it is possible that irritation of the peritoneum was caused by methotrexate traveling down the shunt. Two additional patients with ventriculoperitoneal shunts did not have abdominal pain at any time during the study. Additional grade 1 and 2 adverse events, either unrelated or possibly related to infusions, included headache, diarrhea, dry eye, nasal congestion, cough, gastroesophageal reflux, abdominal pain, sore throat, dysphagia, malaise, fatigue, fever, vomiting, anorexia, chills, back pain, dizziness, and oral mucositis. None of these adverse events were associated with any long-term problem over the duration of follow-up.

Six patients underwent baseline neuropsychological evaluations prior to the planned first cycle. Four of the five patients who received methotrexate infusions underwent follow-up neuropsychological evaluations after the third cycle. In all four patients, there were no significant changes from baseline tests assessing development and adaptive functioning, intelligence, memory, executive functioning, attention, expressive language, or visual and motor function. The fifth patient (patient 7) was removed from the protocol due to disease progression after the third cycle prior to obtaining repeat neuropsychological testing.

The median serum methotrexate level serum methotrexate level was 0.04 µmol/L, and the range was 0.02–0.13 µmol/L. No patient required leucovorin rescue. Median CSF trough methotrexate level was 3.18 µmol/L, and the range was 0.53–212.36 µmol/L. Additional CSF studies for gram stain and culture, cell count, glucose, and protein did not reveal evidence of CSF infection in any patient.

In all five patients who received methotrexate infusions, MRI scans performed after the first cycle, third cycle, and every third cycle thereafter revealed no radiographic evidence of leukoencephalopathy or any damage to the brainstem, cerebellum, or cerebral cortex. Four patients underwent methotrexate cycles without interruption. Despite stable disease, intraventricular methotrexate infusions were stopped in patient 2 after cycle 3 at the request of the patient’s family. Three months later, without further treatment, he had disease progression. With permission of the institutional review board, he underwent reoperation for tumor resection and was re-entered into the study. His postoperative MRI served as a new baseline prior to subsequent cycles.

Both patients with anaplastic ependymoma had progressive disease and were removed from the study after 9 and 3 cycles, respectively. Patient 2 died of progressive disease 20 months after enrollment, and patient 7 died of progressive disease 5 months after enrollment.

**Fig. 3:**
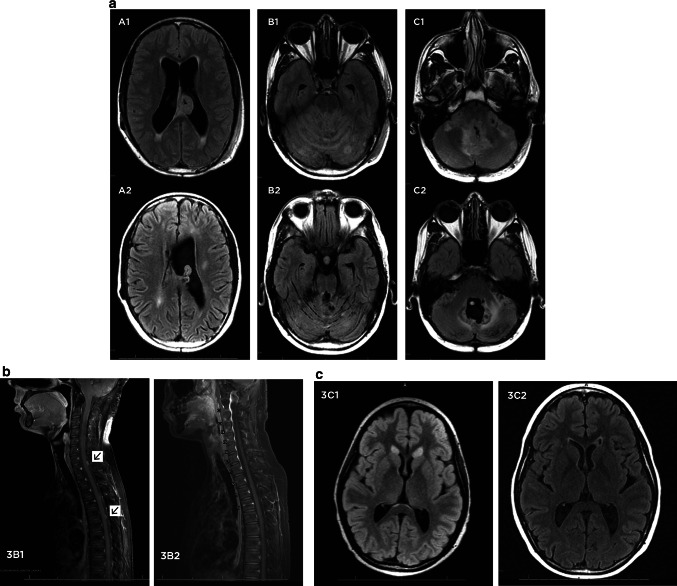
MRI scans demonstrating treatment response in patients 1 and 4. **a** Fluid attenuated inversion recovery (FLAIR) MRI scans in patient 1 at baseline (*top row*, images *A1*, *B1*, and *C1*) and after 15 cycles of intraventricular methotrexate (*bottom row*, figures *A2*, *B2*, and *C2*), 10 months after initiation of the first cycle. There has been a considerable decrease in disease burden in the lateral ventricles, cerebellar folia, fourth ventricle, and cerebellopontine angles. FLAIR sequences are demonstrated because the majority of the tumor burden in the brain was non-enhancing and best visualized on FLAIR. **b** T1-weighted MRI of the spine with gadolinium in patient 4 at baseline (*left*, image *3B1*) and after 15 cycles of intraventricular methotrexate (*right*, image *3B2*). Two small metastatic spine lesions (demonstrated by *arrows*) have disappeared. **c** Axial FLAIR sequences in patient 5 at baseline (image *3C1*) and after 18 cycles (image *3C2*) of intraventricular methotrexate. FLAIR sequences are demonstrated because both were non-enhancing lesions best visualized on FLAIR. There has been a considerable decrease in the lesions, both of which have nearly resolved

All three patients with medulloblastoma had decreased tumor burden after intraventricular methotrexate infusions. Two patients are currently alive (19 and 17 months after enrollment), and one (patient 1) died 25 months after enrollment. At the time of enrollment, patient 1 had massive tumor burden filling the fourth ventricle, coating the cerebellar folia, and involving the pituitary region, lateral ventricles, third ventricles, and thalamus. His disease burden progressively decreased with treatment but never reached the 50 % threshold required for the definition of partial response (Fig. 3a). After cycle 12, his overall disease burden had decreased by 42 %. After cycle 15, he was noted to have slight (<25 %) increase in 3 of 4 target lesions but complete disappearance of the 4th target lesion (Fig. 3b). After cycle 18, one of his target lesions progressed by more than 25 % so he met the definition of progressive disease and was removed from the study.

Patient 4 had disease only involving the spinal subarachnoid space. He had a partial response after cycle 3 that was maintained after cycles 6, 9, and 12. After cycle 12, only a thin line of enhancement remained at the site of a thoracic spine metastasis, and the decision was made to stop therapy. Unfortunately, his thoracic disease progressed on a follow-up MRI scan 3 months after he was removed from the protocol.

Patient 5 had metastatic nodules in the frontal horns of both lateral ventricles. Both nodules progressively decreased in size until, after cycle 12, she met the definition of partial response. Both lesions have continued to decrease in size and have nearly disappeared, and she still meets the definition of partial response after 18 cycles (Fig. 3c). She has been removed from the study but continues to receive intraventricular methotrexate into her fourth ventricle catheter off protocol at her home institution.

Patient 1 had positive CSF cytology in his baseline lumbar puncture and again after the first cycle. Cytology was negative for all subsequent lumbar puncture samples (after cycles 2, 3, 6, 12, 15, and 18) with one exception (after cycle 9). During his first three cycles, all 12 CSF cytology samples from the fourth ventricle (100 %) were positive. Over cycles 4 through 12, only 7 of 36 CSF samples from the fourth ventricle (19.4 %) were positive. From cycles 13 through 18, all 24 CSF samples from the fourth ventricle were negative. The remaining 4 patients who received treatment had negative cytology in all CSF samples from lumbar punctures and from the fourth ventricle.

## Discussion

For children with malignant tumors originating within the fourth ventricle, recurrences most commonly occur at the initial site of disease and/or in the subarachnoid spaces of the brain and spinal cord [4–6]. Ideally, novel treatment approaches should achieve both local and regional tumor control while minimizing systemic drug exposure, as metastatic disease outside of the central nervous system (CNS) is rare. Previous studies in piglets and non-human primates demonstrated that a catheter can be safely placed into the fourth ventricle and that chemotherapy can be infused without causing new neurological deficits or other recognized toxicity [7–10]. These infusions yielded high drug levels in the fourth ventricle as well the spinal subarachnoid space that are maintained for at least 24 h with undetectable or negligible systemic drug levels [7–10].

There are many potential advantages of fourth ventricular chemotherapy administration over infusions via repeated lumbar puncture or VAD placed into the lateral ventricle, both of which have been extensively utilized in previous studies [11–16]. Lumbar punctures are technically difficult in some patients and require sedation in young children. Moreover, infusions via lumbar puncture yield lower and less consistent ventricular drug levels than intraventricular infusions [17, 18]. While catheter placement into the lateral ventricle requires a separate surgical procedure from posterior fossa tumor resection, catheter placement into the fourth ventricle can be performed simultaneously to tumor resection and thereby spare patients an additional operation. Moreover, because lateral ventricle catheters are placed blindly through cerebral cortex, both catheter malposition and cerebral hemorrhage are reported complications [12, 19–21]. Fourth ventricle catheters are placed under direct vision without passing through brain parenchyma, so catheter malposition and hemorrhage are unlikely. Additionally, because lateral ventricle catheters are placed blindly into a ventricle that may be relatively small, some catheter holes may be within the ventricle and others within the cerebral cortex. Infusions may force the infused agent into brain parenchyma, contributing to leukoencephalopathy [12, 22–25]. In contrast, with fourth ventricular catheters, placement of the catheter under direct vision ensures that no catheter holes are within brain parenchyma. When placing fourth ventricular catheters and ventricular access devices, great care must be taken to secure the catheter in a way that ensures that it stays oriented parallel to the long access of the fourth ventricle to avoid brainstem injury. The catheter must be secured well to bone or fascia to ensure that it does not migrate.

It is important to note that many of the complications of lateral ventricle catheters described above can be minimized by using frameless stereotaxy to ensure that a catheter placed into the lateral ventricle is in the ideal position at the foramen of Monro. Moreover, fourth ventricle ventricular access devices may not be the ideal way to distribute drugs to metastatic lesions in certain patients depending on the anatomic location of the lesions. For example, some patients may potentially achieve higher drug levels in the lateral and third ventricles with a lateral ventricle catheter than with a fourth ventricle catheter.

In this study, serum methotrexate level 4 h after infusions was consistently low (median 0.04 µmol/L). Low systemic drug levels are favorable when treating malignant brain tumors that only rarely spread outside of the central nervous system (CNS), as high systemic drug levels can cause toxicity to organs unaffected by cancer. In order to avoid additional reservoir taps besides those required for treatment, only trough CSF methotrexate levels were measured in this study. Trough CSF methotrexate levels were nearly 80-fold higher (median 3.18 µmol/L) than serum levels. It would be expected based upon prior animal studies that peak methotrexate levels, which were not measured in the current study, would likely have been several orders of magnitude higher than trough levels [7, 9, 10]. Ideally, methotrexate distribution would be assessed by lumbar spine CSF, but these were not performed to avoid lumbar punctures that would not have affected the patients’ plan of care.

The primary objective of this study was to demonstrate that a chemotherapeutic agent may be infused via an implanted catheter into the fourth ventricle, next to the brainstem and cerebellum, without causing neurological toxicity. This objective was achieved based upon both radiographic and clinical criteria. Radiographically, no patient demonstrated leukoencephalopathy or other evidence of brain injury. Clinically, 240 consecutive infusions of methotrexate were administered in five patients without any new neurological deficits or other serious systemic toxicity. The patients who received the most infusions, patients 1 and 5, received 18 cycles (64 infusions) over approximately 1 year without any toxicity radiographically or clinically. No patient had any changes in neuropsychological examinations after 3 cycles of chemotherapy. Of course, neuropsychological changes may take years to develop after treatment, and long-term neuropsychological outcomes were not assessed by this pilot study.

Response to treatment in all three patients with medulloblastoma was an unexpected positive finding, as this pilot study used a low dose (2 mg) of methotrexate to minimize the risk of toxicity. The outcomes of these three patients demonstrate that infusions into the fourth ventricle can lead to treatment response not only in the posterior fossa but also rostral to the infusions (in the lateral ventricles) and caudal to the infusions (in the spinal subarachnoid spaces). Unfortunately, both patients with ependymoma exhibited progressive disease after 9 and 3 cycles, respectively, and were therefore removed from the study. Historically, ependymoma is less chemosensitive than many other malignant brain tumors, and we hypothesize that either methotrexate is not the ideal drug for these patients or that 2 mg is not an adequate dose. The concept of local delivery for ependymoma remains appealing, as recurrent ependymoma is very difficult to treat effectively with currently available systemic chemotherapy regimens.

In summary, this pilot trial has demonstrated that low-dose methotrexate can be infused into the fourth ventricle in patients with recurrent, malignant tumors without causing recognized neurological deficits or other significant toxicity. Partial responses in several patients offer proof of principle that intraventricular chemotherapy may be effective in treating malignant brain tumors, as the overwhelming majority of previous trials utilized simultaneous systemic chemotherapy. This trial will form the basis for future trials to explore the safety and efficacy of higher doses of methotrexate, as well as infusion of additional agents into the fourth ventricle, in an effort to expand treatment options and improve outcomes for patients with malignant posterior fossa tumors.
